# Healthcare access and perceived value of liver screening among people experiencing homelessness and substance use disorders: a qualitative study

**DOI:** 10.1186/s12913-025-13809-z

**Published:** 2025-12-06

**Authors:** Nikolaos Mylonas, Laura Hazeldine, Maria Walsh, Nicole Bretana, Chris Daly, Martin Prince, Stephen J. Kaar

**Affiliations:** 1 Greater Manchester Mental Health NHS Foundation Trust, Manchester, UK; 2 Division of Nursing, Midwifery & Social Work, Faculty of Biology, Medicine and Health, University of Manchester, Manchester, UK; 3 Manchester University NHS Foundation Trust, Manchester, UK; 4 Division of Psychology and Mental Health, School of Health Sciences, Faculty of Biology, Medicine, and Health, University of Manchester, Hathersage Road, Manchester, M13 9WL UK

**Keywords:** Homelessness, Liver disease, Vibration controlled transient elastography, Drugs and alcohol

## Abstract

**Background:**

People experiencing homelessness face multiple barriers to healthcare and are at increased risk of advanced liver disease, which often remains undiagnosed. This study explored the acceptability and perceived value of a novel liver screening pathway using Vibration Controlled Transient Elastography (VCTE), embedded within an assertive outreach drug and alcohol service.

**Methods:**

Semi-structured interviews were conducted with service users experiencing homelessness (*n* = 12) post-VCTE. Questionnaires assessing substance use history, social networks, and readiness to change were used to characterise the participants. Qualitative data was analysed using thematic analysis.

**Results:**

Participants’ scores indicated high levels of readiness to change and limited social networks. Three interrelated themes were identified in the semi-structured interviews. First, participants reported co-occurring vulnerabilities—including polysubstance use, housing precarity, and trauma—which, in combination with previous negative experiences and stigma, contributed to reduced engagement with conventional healthcare services. Second, the provision of VCTE within an assertive outreach drug and alcohol service was perceived as a more accessible and acceptable alternative to mainstream pathways. Third, the screening encounter was experienced as a reflective moment, with several participants describing increased awareness of liver health and, in some cases, enhanced motivation to modify risk behaviours.

**Conclusions:**

Liver screening embedded within an outreach-based drug and alcohol service was acceptable for users of those services and, for some, represented an opportunity for reflection and harm reduction. Findings support the integration of non-invasive liver screening into services for people experiencing homelessness to improve early detection and engagement with care.

**Supplementary Information:**

The online version contains supplementary material available at 10.1186/s12913-025-13809-z.

## Background

People experiencing homelessness face numerous barriers to accessing healthcare services and are often marginalised and stigmatised [1–3]. Liver pathology, which includes alcohol-related liver disease and viral hepatitis (hepatitis B and C virus), can progress to fibrosis, end-stage liver cirrhosis and cancer [4, 5] and is responsible for much of the early mortality seen in this cohort [6, 7]. The prevalence of liver disease is high in people experiencing homelessness [8–10], yet they typically receive a diagnosis when the pathology is advanced and irreversible, as the condition often remains asymptomatic in its initial stages [11, 12]. Early detection of liver disease among this cohort is paramount for reducing morbidity and premature mortality [13].

Non-invasive portable liver scanning has improved the ability to detect advanced liver disease at an earlier stage [11, 14]. Vibration Controlled Transient Elastography (VCTE) is a quick, non-invasive screening test for fibrosis and cirrhosis detection [15]. The device quantifies liver stiffness by using a probe that generates a shear wave travelling through the subcutaneous tissue into the liver [16]. The device’s algorithm measures the wave’s velocity and converts it into an estimate of liver stiffness, expressed in kilopascals (kPa) [17]. A kilopascal is a unit of pressure in the International System of Units (SI), equal to 1,000 pascals. The pascal, named after Blaise Pascal (1623–1662), is defined as one Newton per Square Metre, or in SI base units, one kilogram per metre per second squared [18]. Research indicates that the optimal cut-off value for diagnosing advanced fibrosis or cirrhosis is around 14 kPa, although this varies according to the aetiology of liver disease [15]. Fibrosis scores are typically staged from F0 to F4, where F0 indicates no fibrosis, F1–F3 indicate increasing levels of liver scarring, and F4 is consistent with cirrhosis [19]. Measurements above this threshold are associated with a 90% probability of cirrhosis [20]. A study of case identification among people experiencing homelessness with hepatitis C virus (HCV) in Boston [21], USA found that 15.8% of them had advanced fibrosis (F3 and F4), while a study on a cohort of people experiencing homelessness in England [12] found that 17% of them had cirrhosis (F4).

Although there is growing literature on the utility of early detection of fibrosis in individuals with alcohol use disorder [12, 22, 23], there is limited research exploring the factors facilitating the acceptability and perceived value of VCTE when delivered in community addiction services, particularly those targeting people experiencing homelessness. Most existing studies have focused on clinical outcomes and diagnostic accuracy, with less attention paid to the experiences, expectations, and responses of service users themselves. Given the well-documented barriers this population faces in accessing mainstream healthcare—including stigma, structural disadvantage, and competing priorities—there is a pressing need to evaluate how novel, assertive outreach-based screening interventions are received and whether they may contribute to improved engagement with health services [24–27].

This study pursued four specific aims: (1) to identify barriers to healthcare access, particularly in primary and acute care settings; (2) to examine service users’ experiences of the new liver screening pathway, including perceived accessibility, communication, and delivery; (3) to understand how individuals interpreted the procedure and its results; and (4) to explore the impact of the screening on participants’ motivation to reduce harm, seek treatment, or access further care. In order to further characterise the readiness to change and the social networks of our participants, we used relevant questionnaires. The findings aim to support the design of liver screening interventions that are accessible, acceptable, and responsive to the needs of people experiencing homelessness within assertive outreach drug and alcohol services.

## Methods

### Design

This study examined service users’ experiences of accessing both the new pathway and existing healthcare services. Questionnaires were used to describe participants’ substance use patterns, readiness to change, and social networks. Semi-structured interviews explored their experiences of acute and primary physical healthcare in greater depth, as well as their perspectives on the potential of VCTE to encourage harm reduction and further engagement with services.

### Setting

The pathway was set up as a collaboration between a local government body and a National Health Service-based mental health organisation in North West England, UK, to increase the early detection of liver disease in people experiencing homelessness and substance use disorders. This cohort typically has limited engagement with primary care services, and detection of advanced liver disease tends to happen at a late stage during an acute hospital admission. The pathway was implemented within a specialist drug and alcohol outreach service for people experiencing or at risk of homelessness and aimed to perform VCTE on all individuals referred for substance use disorders at the point of assessment. The test is performed by a trained substance misuse nurse. Service users with a score of fibrosis F4 are referred to the hepatology department of the local acute hospital. All service users are offered harm reduction advice based on their results and individual needs.

### Inclusion and exclusion criteria

Eligible participants were service users referred to the liver fibrosis screening pathway between January and May 2025 who:

- Had a current substance use disorder.
- Were homeless or at risk of homelessness.
- Were able to provide informed consent.
- Could understand and converse in English.
- Had an identified professional or keyworker available to support recruitment and risk assessment.

Individuals were excluded if they:

- Could not provide informed consent.
- Did not have a history of substance use disorder.
- Were not homeless or at risk of homelessness.

### Materials

The EDIF Screening Questionnaire (See additional file 1) explored sociodemographic characteristics, drug and alcohol use history, self-reported mental health diagnosis, current housing status, history of incarceration and self-reported disability.

The Readiness to Change Questionnaire (Treatment Version) (RCQ-TV) (Revised) [28] is a 12-item instrument used to measure the “stage of change” reached by individuals dealing with alcohol use disorder. It is based on the transtheoretical model of change developed by DiClemente and Prochaska [29]. The instrument’s items include four statements for each of the three stages of change (‘Precontemplation’, ‘Contemplation’, ‘Action’). Although this questionnaire is popular in research studies measuring the motivation of people experiencing substance use disorders [28], it is important to note that it has certain limitations when used in a cohort of people experiencing homelessness. The instrument adopts an individualist lens of ‘readiness to change’ reducing alcohol use to the relationship an individual has with the substance. As Harris ([26], p. 2) argues, there is a necessity to have “a shift of focus from individual practice and drug supply as crucial points of intervention, to the systems and structures that can render health care access problematic for the most marginalised” [26]. However, an assessment of participants’ scores using this instrument was judged as potentially informative as it was hypothesised that it might challenge the commonly held assumption that this cohort ‘lacks motivation’ to address their substance use disorders and access healthcare services [30].

The Readiness to Change Questionnaire (Treatment Version) (RCQ-TV) (Revised & Adapted) [31] is an identical 12-item instrument which was adapted for participants with opiate use disorder.

The Important People Initial Interview [32] was used to assess participants’ social networks and the role of significant others in relation to their substance use. This structured questionnaire captures information on the number and type of important people in a participant’s life, the frequency and quality of contact, and the extent to which these individuals support or discourage substance use and treatment engagement.

The topic guide (See additional file 2) was informed by the NICE (National Institute for Health and Care Excellence) Guideline NG214 on integrated health and social care for people experiencing homelessness [33] and was co-produced with an expert by experience. Topics were also discussed during a patient and public involvement and engagement event with people experiencing substance misuse or/and homelessness (11th June 2024) and covered the context of referral to the outreach drug and alcohol service, experiences of engagement with primary and acute care, social network support in engagement with services, experiences of receiving care for liver problems, experiences of VCTE, and reaction to feedback from the result.

The Participant Information Sheet and the Consent Form were reviewed by two experts-by-experience. The research ethics committee approved all the materials.

### Recruitment

Service users who completed the VCTE scan were initially approached by their clinical team, and if they were willing to participate, an appointment was arranged to obtain written consent. All participants had sufficient time to go through the participant information sheet with the aid of the researcher and had the opportunity to ask questions. All participants received a £10 shopping voucher as compensation for their time.

### Data collection

Qualitative data were collected between February and May 2025. Following written consent, the interviews were audio-recorded using encrypted devices provided by the NHS organisation that sponsored the study and transcribed verbatim using the Microsoft Teams ‘Record and transcribe’ function. The transcripts were later reviewed by two researchers for accuracy. Questionnaire data were collected in paper format and then transcribed into a Microsoft Excel spreadsheet. Out of 12 participants, one declined to complete the Important People Initial Interview questionnaire, so their data are not included in the analysis of the results of this questionnaire.

### Data analysis

Descriptive statistics of questionnaire data were calculated using the R programming language [34]. Interview transcripts were coded by two researchers using NVivo 14 software. The researchers coded three out of 12 transcripts together and then coded independently the rest. Qualitative data were analysed using thematic analysis [35]. Coding combined inductive theme development with a deductive framework derived from the NICE Guideline NG214 on integrated health and social care for people experiencing homelessness [33]. This guideline was selected for its population-specific relevance and its emphasis on coordinated, trauma-informed, and person-centred approaches to care. The guideline provides a comprehensive understanding of the challenges people experiencing homelessness face in engaging with health services, and it informed both code development and the interpretation of themes in relation to service user experiences and service provision. The NICE Guideline NG214 ([33], p. 7) states: “Barriers to access and engagement with preventive, primary care and social care services can mean that problems remain untreated until they become very severe and complex. These barriers include stigma and discrimination; lack of trusted contacts; fragmented, siloed and rigid services; strict eligibility criteria; and lack of information sharing and appropriate communication.” Relevant interview extracts were coded as “experiences of healthcare services” and “stigma”. The guideline ([33], p. 24) also recommends: “Offer collaborative, assertive outreach to start and maintain engagement with health and social care for people experiencing homelessness with coexisting severe mental health and drug or alcohol treatment needs”; this guided the coding of extracts the coding of extracts referring to outreach provision (“Outreach model”). Relevant extracts regarding offering harm minimisation as part of outreach ([33], p. 24) and the service users’ motivation were coded as “Feedback post-scan” and “Service user motivation”.

### Ethics

This study was approved by the Health Research Authority (HRA) and Health and Care Research Wales (HCRW) on 7th November 2024 (REC reference: 24/SW/0089). All participants signed a written consent form to participate in this study. References to specific services and locations have been redacted.

### Reflexivity

All the authors of this article have experience in delivering clinical care and/or conducting research with people experiencing homelessness and substance misuse. Mitigating potential bias based on previous experiences, we engaged in regular team discussions during the data collection and analysis stages.

## Results

### Participant demographics and substance use profiles

Only one service user participant self-identified as female. With the exception of one individual of British Asian ethnicity, all participants identified as White and heterosexual. The majority of participants reported daily, excessive alcohol consumption, averaging 20.3 alcohol units per day, and an average duration of alcohol dependence of 17.5 years. Most participants also reported daily use of heroin and crack cocaine, with an average history of unregulated drug use approaching 15 years. All but one participant used at least one type of unregulated drug. Six out of the 12 participants disclosed current or previous use of intravenous routes of administration. The most commonly reported mental health diagnoses were depression and anxiety (*n* = 7), while two participants reported a diagnosis of paranoid schizophrenia. These diagnoses were also mentioned in the participants’ medical records. Only four participants were receiving pharmacological treatment for mental health conditions at the time of the study. Most were residing in hostels designated for people experiencing homelessness, and one participant reported rough sleeping during the recruitment period. Participant demographic and substance use characteristics are summarised in Table 1.

**Table 1:** Characteristics of the cohort

*n*	12
Median age (SD, range)	43 (8.2, 34–60)
Gender (n/%)	
Male	11 (91.7)
Female	1 (8.3)
Ethnic Group/Race (n/%)	
White British	10 (83.3)
White Other	1 (8.3)
British Asian	1 (8.3)
Sexual Orientation (n/%)	
Heterosexual	12 (100)
Alcohol Use Frequency (n/%)	
Daily	8 (66.7)
4 days a week	1 (8.3)
Monthly or less	1 (8.3)
Never	2 (16.7)
Daily Consumption Alcohol Units Mean (SD/range)	20.3 (17.6, 0–50)
Years of alcohol dependence Mean (SD/range)	17.5 (17.1, 0–48)
Type of unregulated drug use (n/%)	
Heroin and crack cocaine	8 (66.7)
Cannabis	1 (8.3)
Crack cocaine	1 (8.3)
Cocaine	1 (8.3)
No unregulated drug use	1 (8.3)
Frequency of unregulated drug use (n/%)	
Daily	7 (58.3)
1–4 days a week	1 (8.3)
Monthly	1 (8.3)
Not in the last 6 months	3 (25)
Years of unregulated drug use Mean (SD/range)	14.9 (12.2, 0–36)
Routes of unregulated drug administration (n/%)	
Intravenous	6 (50)
Inhaled	4 (33.3)
Nasal	1 (8.3)
Not applicable	1 (8.3)
Mental Health Diagnosis (n/%)	
Paranoid schizophrenia	2 (16.7)
Depression and anxiety	7 (58.3)
None	3 (25)
Takes mental health medication (n/%)	
Yes	4 (33.3)
No	8 (66.7)
Current housing status (n/%)	
Mainstream housing	1 (8.3)
Short-term hostel accommodation	8 (66.7)
Sofa surfing	2 (16.7)
Rough sleeping	1 (8.3)
Prison (n/%)	
Has never been in prison	8 (66.7)
Has been in prison earlier than 12 months ago	3 (23)
Has been in prison in the last 12 months	1 (8.3)
Disability (n/%)	
Hearing	1 (8.3)
Mobility	1 (8.3)
Memory or Learning	1 (8.3)
Progressive Conditions and Physical Health	1 (8.3)
None	8 (66.7)

### Fibrosis, hepatitis C status and readiness to change

Three (25%) participants had a fibrosis stage higher than F0, and one had newly diagnosed F4 fibrosis (cirrhosis) (8.3%). Four participants were found to have been infected with HCV in the past, but they did not have an active infection. Regarding readiness to change substance use behaviour, six participants were categorised in the “Contemplation” stage of change and six in the “Action” stage. This finding indicates that the stigmatising assumption that individuals experiencing homelessness and substance use disorders often lack motivation to address their substance use is not warranted for our sample. Liver health, HCV status and readiness for change stage are summarised in Table 2.

**Table 2:** Participants’ results reflecting Readiness to Change Questionnaire, VCTE and hepatitis C virus status

*n*	12
Readiness to Change Questionnaire Score (Revised or Revised & Adapted) (n/%)	
Pre-Contemplation	0
Contemplation	6 (50)
Action	6 (50)
Stiffness of Liver (kPa) Mean (SD/range)	6.4 (4.3, 3.6–19.2)
Fibrosis Score (n/%)	
F0	9 (75)
F1	2 (16.7)
F2	0
F3	0
F4	1 (8.3)
CAP Score (db) Mean (SD/range)	232.7 (55.9, 156–354)
Hepatitis C Virus Status (n/%)	
Never infected	7 (58.3)
HCV antibody detected	4 (33.3)
Not known	1 (8.3)

### Characteristics of social network members and attitudes toward substance use

The majority of individuals identified in the Important People Initial Interview (IPI) were either parents or children of the participants. Most participants reported frequent contact—defined as at least once per week—with these individuals. Only 9.1% of the individuals identified were reported to be heavy alcohol or drug users, while 63.7% were characterised as abstinent from both alcohol and unregulated drugs. In terms of responses to substance use, 36.4% of the identified individuals were described as not accepting the participant’s drinking or drug use, and 18.2% had reportedly left or asked the participant to leave due to their substance use. Despite these responses, 81.8% of the individuals were perceived by the participants as strongly supportive of their engagement with drug and alcohol treatment. The full results of the IPI are summarised in additional file 3.

### Semi-structured interview data

Drawing on qualitative interviews, three interrelated themes were identified. First, participants described overlapping vulnerabilities—including high-risk poly-substance use, housing instability and trauma—which were associated with difficulties engaging with health services, often marked by limited access and perceived stigma. Second, the delivery of VCTE within an outreach-based drug and alcohol service was viewed by the users of that service as a more accessible and acceptable alternative to mainstream provision. Finally, the screening itself was interpreted by participants as both a harm reduction tool and a moment of reflection, eliciting varied emotional responses and, in some cases, motivating intentions to reduce risk behaviour.

#### Theme 1. Multiple vulnerabilities intersecting with health service engagement and experiences

Participants reported high-risk alcohol consumption and, in most cases, concurrent use of heroin and crack cocaine. Participants reported living in precarious housing arrangements for years. When discussing the circumstances of their lack of stable housing, they described substance misuse, exploitation, and breakdown of relationships with family members or partners. One participant described how he was referred to the rough sleeper drug and alcohol team in his area.

*I evacuated my flat because I was getting bullied and ended up on the street and then the rough sleepers team got in touch with me. So*,* they then I got referred to drug and alcohol team. (Participant 9).*

Participants frequently described their use of substances in the context of having experienced traumatic events, but also traumatic experiences that were a consequence of high-risk substance misuse. One participant mentioned:

*Because you shut things off. Things you don’t want to remember (Participant 1)*

These interconnected vulnerabilities were associated with the de-prioritisation of engagement with health services and increased risk of liver disease. Participants reported having difficulties in achieving consistent engagement with both primary and acute healthcare services in spite of the numerous reported long-term conditions and accidental injuries. Interviews described having limited access to healthcare interventions as an outcome of prioritisation of trying to secure funds for substance use. This was mentioned primarily by participants referred for opiate use disorder. Despite identifying some individuals in their lives as “important people”, participants did not expect to receive concrete support from them in engaging with services. However, the main barriers identified by our participants in their engagement with healthcare services were previous negative experiences and stigma.

Most participants reported having very limited engagement with their primary care provider and described finding it difficult to access care when they have no fixed abode or a limited ability to register and use digital platforms to book appointments. One service user, for example, mentioned that he is struggling to attend when appointments are booked.

*He [participant’s primary care doctor] said I’ve beaten the world record and missed that many appointments. (Participant 3)*

While another service user mentioned that primary care is difficult to access when digital devices or smartphones are required.

*I don’t know to be honest. Find it hard to access. That’s all for most people. Especially if they can’t use technology. (Participant 8)*

Nevertheless, some participants reported having some positive experiences in their encounters with primary care, characterised by empathy and understanding.

*My doctor does understand. He is going to sort out my prosthetic leg as well. (Participant 10)*

The experiences of the participants in acute hospital settings were reported as being overwhelmingly negative, prolonging feelings of distrust and leading to delays in care-seeking. The primary focus upon discussing these experiences was, first, feeling stigmatised for substance misuse and, second, unsafe discharges. In some instances, participants reported feeling discriminated against by clinicians working in acute hospital settings.

*They just look at you a little bit different*,* you know what I mean? Don’t get me wrong*,* some of them are really nice. Some are really nice. You get some of them that are just*,* you know*, basically can’t be ar**d. “I know you are in pain,* but we will sort it when we sort it.” (Participant 5)*

One service user described a case of unsafe discharge in detail:

*So*,* my housing worker was trying to get me a place. But there was nothing she could do […] she came to the hospital to see me every day and she kept me in there for like extra week*,* you know. She told the nurses like “should we get him somewhere to stay”. And they just kicked me out on the streets*,* I didn’t even get a hostel. Got kicked out onto the streets. (Participant 7)*

However, other participants mentioned that feelings of anxiety and shame prevent them from asking for help. For example, one participant described how he hesitates to reach out to services for a condition [alcohol use disorder] he sees as self-inflicted:

*“You know*,* I’m ashamed. I put myself in this position*,* and then I’m asking others for help. (Participant 12)*

#### Theme 2. Assertive outreach and person-centred communication as engagement tools

In the context of inconsistent engagement with mainstream health services, the offer of VCTE from an outreach-based drug and alcohol service aimed at people experiencing homelessness was perceived as a more accessible option for the early detection of fibrosis. An assertive outreach approach and person-centred communication before and after the procedure of the scan were identified as facilitators of engagement.

The offer of the screening on an outreach basis was deemed very important for the successful completion of the examination. All participants were either scanned at their residence or were supported by the drug and alcohol outreach staff to reach a local community venue where the scan was performed. Participants discussed how their keyworker supported them by booking the appointment, arranging their transportation to the service building or accompanying the nurse in conducting a home visit in cases when the scan was performed in participants’ temporary accommodation.

*Well*,* she picked me up in the car. She walked me down*,* and she just basically said they’re going to check you out. So that was brilliant. I went in*,* they did the Fibro Scan*,* which didn’t take very long at all. I was just about 5 minutes*,* and I’ve got the results there [showing the document with his results]. (Participant 3)*

Others mentioned that they would probably not attend the appointment if they did not have the support of the outreach staff. When asked for the reasons for that, one service user mentioned:

*Because I am just not used to going out and meeting new people. (Participant 12)*

The interactions between service users and staff before the scan were considered key for engaging a cohort of individuals who often have negative experiences with health services. Service users mentioned how VCTE was portrayed as a ‘new’ method to detect liver problems and that attending the appointment with the outreach staff was helpful in dealing with feelings of anxiety about possibly negative results. All participants, however, mentioned that they did not hesitate to accept the procedure.

*She [staff] said it was a new invention sort of thing. A new technique they were using to test people’s livers. (Participant 9)*

*I was nervous going in there*,* think they are gonna give me some real bad news there. That’s what I thought. (Participant 5)*

*He [his keyworker] did talk all about it step by step. (Participant 12)*

Most participants were impressed with how quick the procedure was compared with the usual waiting time to receive results for healthcare investigations. Some interviewees referred to the person-centred approach that the staff used to communicate the result to them. In cases where the fibrosis score was low, participants mentioned that both the nurse who performed the scan and their outreach worker emphasised caution and offered harm reduction advice, while in cases of a higher stiffness score, they were prompted to aim for a lower score in a future screening following a plan to reduce consumption.

*Well*,* I don’t how long*,* it’s pretty much instant. They can see straight away what’s happening with your liver. (Participant 2)*

*I thought I’d be dead now be honest. No*,* I did*,* but she said that obviously chill out a bit*,* basically*,* in her own words*,* “calm it down a bit. You’re lucky” that’s what she said. (Participant 3)*

#### Theme 3. Perceived value of VCTE as a harm reduction tool

As some participants disclosed experiencing anxiety before the appointment, it was deemed useful to explore their reaction when they received the results. In cases where the fibrosis score was low, participants reported feeling relief and surprise that despite high-risk substance misuse for long periods of time, their liver had not been damaged. Most participants with a low fibrosis score mentioned that they were expecting a much higher score.

*Look at that [showing his results]*, second one down. A little bit damage but not f***** up. (Participant 1)

*Yeah*,* it did surprise me. Because I thought my liver was going to be far gone. That’s why I was surprised that it’s not that far gone. It’s not irreversible. (Participant 9)*

Significantly, some service users mentioned experiencing feelings of guilt or being spared that their liver was still healthy in spite of high-risk behaviours when compared to family members and younger individuals who face significant health problems.

*Because I’ve damaged my body with every single drug under the sun. And there’s nothing wrong with it. The family is all I’ve got left with me and they’re dying. It isn’t fair. Life is not fair. (Participant 6)*

*There’s a young lad here*,* he is only 23-year-old or something like that. He has got cirrhosis of the liver. That shocked me. Not that I can believe it. He needs a transplant*,* if he doesn’t get that he is dead. I sat there and I was like ‘wow’. How lucky have I been from what I have done over the years. (Participant 5)*

Participants were asked whether the VCTE procedure was an opportunity to discuss risk behaviours and increase their motivation to reduce harm. Some interviewees mentioned that a high fibrosis score was perceived as a warning to address their substance use disorder in a timely manner, while others mentioned that although they recognised the risk, they would not consider a detoxification and rehabilitation placement until other problems, such as housing, had been addressed.

*Frightened me. You know what I mean. Yeah*,* because my dad’s got alcohol liver disease on his death certificate. I don’t want to go that way. (Participant 9)*

I’m thankful for that scan. It give me a kick up the a*** basically because I thought,* you know*,* I’m so*,* so lucky. I am just so thankful that I’ve got this opportunity (Participant 5)*

*No*,* it’s pointless. It’s pointless. Maybe when I get my own flat further down the line a bit*,* may be considered*,* but at the moment*,* no chance. (Participant 3)*

## Discussion

This study explored experiences of healthcare access and perceived value of liver screening within a VCTE pathway embedded in an assertive outreach drug and alcohol service for people experiencing homelessness. Specifically, it examined: (1) barriers to healthcare access, particularly in primary and acute care settings; (2) how individuals experienced the screening, including perceived accessibility and communication; (3) how the procedure and its results were interpreted in the context of their previous engagement with healthcare services; and (4) whether and how the intervention influenced motivation to reduce harm or seek further care. Using the NICE NG214 guideline [33] as a deductive coding framework, this study demonstrates how participant perspectives reflect and reinforce many of the principles outlined in this document for integrated, trauma-informed care for people experiencing homelessness. The findings contribute to a growing body of literature indicating that non-invasive liver screening tools such as VCTE are both feasible and acceptable in community settings and may function as tools for promoting harm reduction and engagement with health services [22, 36, 37].

Participants had, in general, a long history of substance use disorders, yet appeared motivated to address the problem in line with previous research [38]. All participants were grouped in either the ‘contemplation’ or the ‘action’ stage of the two versions of the ‘Readiness to Change Questionnaire’, indicating that even when assessed with an instrument that adopts an individualist understanding of substance use, our participants indicated high levels of motivation. This finding highlights the need to explore further contextual and structural aspects of healthcare access beyond reductionist conceptualisations of ‘individualised responsibility’ in care-seeking [26]. Most of the people our participants identified as important to them do not seem to meet the criteria for substance use disorders, or do not use any substances. One possible explanation is that participants may not regard peers with substance use disorders as “important people” within their social networks. However, experiences of isolation and limited social networks frequently reported in this population could be another factor, given that most participants mentioned their parents or their children [39]. There was no mention of social network support in accessing services, despite some evidence that peer referral is a promising mechanism for case identification of liver disease in people experiencing homelessness and alcohol use disorder [40]. Similarly, the role of ‘advanced peer support worker’ has been utilised in promoting care engagement of individuals experiencing homelessness and who are diagnosed with HCV infection [41].

Similar to previous studies that reported high acceptance of community-based VCTE in homeless or high-risk populations [42], all participants in this study accepted the offer of a liver scan. The accessibility of the intervention was enhanced not only by its location within a drug and alcohol service but also by the outreach strategies employed by staff, such as accompanying clients to appointments or conducting home visits with a portable device. These findings are consistent with earlier calls for the expansion of liver screening to primary care settings [43], highlighting, however, that people experiencing homelessness are more likely to access such screening through an outreach-based community drug and alcohol service aimed at this cohort, in light of challenges they face in engaging with their primary care provider.

Some participants reported feeling nervous before the scan, particularly about receiving potentially serious results. This indicates the value of incorporating pre-scan conversations that not only explain the procedure but also prepare individuals emotionally, particularly those with experiences of trauma or low prior engagement with healthcare and who are often blamed for their health conditions [44–46]. Such adjustments could enhance the psychological safety of the procedure and improve its acceptability further, in line with trauma-informed care principles [47]. While some participants anticipated severe liver damage, the majority were surprised to receive low fibrosis scores. This elicited complex emotional reactions, ranging from relief to disbelief and even guilt—particularly in cases where participants knew younger peers or relatives who had died of liver disease. These findings align with prior literature suggesting that people with liver disease often lack awareness of liver damage until advanced stages [48], and that non-invasive screening can serve as a visible, tangible moment of realisation [49].

Although there is evidence that knowledge of liver fibrosis can increase engagement with drug and alcohol services and reduce high-risk alcohol consumption [22], there is limited research on the potential of liver screening to promote harm reduction behaviour change for people experiencing homelessness. Most participants in our study mentioned that the communication of the results and the conversation with the nurse who performed the screening were deemed beneficial to promote a decrease in risk behaviour. Although all participants were either in the ‘contemplation’ or ‘action’ stage of change regarding their substance use, several noted that addressing their substance use would remain challenging as long as their precarious housing situations persisted. This finding reflects previous research indicating that motivation is shaped by both individual insight and structural constraints, such as lack of housing, fragmentation of care, and difficulty navigating multiple care providers [50].

Findings highlight the importance of delivering interventions in ways that minimise barriers to access and engagement. Traditional models of healthcare delivery, often built around hierarchical and inflexible pathways, are poorly suited to the needs of people facing exclusion or trauma, and can actively deter them from seeking timely care [51]. By offering liver screening in familiar, supportive settings, services can create more immediate and meaningful opportunities for individuals to reflect on their health and consider further support. In this context, embedding VCTE within services already offering psychosocial support increases the likelihood that individuals will accept screening, engage with their results, and reflect on their health. This approach is consistent with the NICE Guideline NG214, which emphasises coordinated, multi-agency responses that bring together housing, healthcare, and outreach-based addiction treatment to improve outcomes for people experiencing homelessness [33]. As other studies have shown, the success of such interventions relies on the design of the care pathway itself, particularly when services are co-located and offer flexible, outreach-based support [36, 52, 53].

To enhance the effectiveness of transient elastography as both a screening and harm reduction intervention, services should consider:

- Pre- and post-scan sessions with keyworker to mitigate anxiety and provide structured interpretation of results.
- Staff training in trauma-informed communication and motivational interviewing.
- Integration of VCTE outreach into housing, mental health, and HCV treatment pathways, recognising that readiness to change is contingent on stability and support.
- Involving supportive family members and peers to enhance engagement with care pathways.

## Strengths and limitations

This study adds to the limited qualitative literature exploring service user experiences of VCTE in drug and alcohol services for people experiencing homelessness. The study is limited by its small sample size, lack of gender and ethnic diversity, which may restrict generalisability. Another limitation is that our sample consisted of individuals with at least a minimal level of engagement with the drug and alcohol assertive outreach service. This service is designed to reach people who struggle to access traditional models of care, but we did not include individuals who had not engaged with any service. Future research should include longitudinal follow-up to assess behavioural outcomes and explore how liver screening interacts with other components of integrated care.

## Conclusions

This study found that VCTE was acceptable and accessible when delivered within a community-based addiction service for people experiencing homelessness. Participants valued the outreach approach and support before and after the screening. The scan prompted reflection on health, and for some, it was perceived as increasing motivation to reduce harm. These findings support the integration of non-invasive liver screening into community drug and alcohol services as a means of improving early detection and engagement in care.

## Supplementary Information

Below is the link to the electronic supplementary material.

Supplementary Material 1: Additional file 1. Screening Questionnaire. Questionnaire that explore sociodemographic characteristics, drug and alcohol use history, self-reported mental health diagnosis, current housing statues, history of incarceration and self-reported disability.

Supplementary Material 2: Additional file 2. Interview Topic Guide. The topic guide that was used for the interviews.

Supplementary Material 3: Additional file 3. Important People Initial Interview Results. Table detailing the results of the Important People Initial Interview instrument.

## Data Availability

Data from semi-structured interviews cannot be publicly available due to privacy and confidentiality concerns. For enquiries about accessing the data, please contact Dr Stephen Kaar, email address: Stephen.kaar@manchester.ac.uk.
