# Supplementary material for: Healthcare access and perceived value of liver screening among people experiencing homelessness and substance use disorders: a qualitative study
Source: BMC Health Serv Res. 2025 Dec 6;25:1588. doi: 10.1186/s12913-025-13809-z (PMC12690787; doi:10.1186/s12913-025-13809-z)
Supplement: Supplementary file 1 — Supplementary Material 1: Additional file 1. Screening Questionnaire. Questionnaire that explore sociodemographic characteristics, drug and alcohol use history, self-reported mental health diagnosis, current housing statues, history of incarceration and self-reported disability. [file 12913_2025_13809_MOESM1_ESM.pdf]

NHS Greater Manchester Mental Health NHS Foundation Trust

# Additional file 1. Screening Questionnaire

***Participant Identification Number***

| | | | | |
|---|---|---|---|---|
| | | | | |

Date Click or tap to enter a date.

**Age** Click or tap here to enter text.

**Gender**

| | |
|---|---|
| Male | |
| Female | |
| Other | |

**Ethnicity**

| | |
|---|---|
| White British | |
| White Other | |
| Black (African or Caribbean) | |
| Asian | |
| Black British | |
| Mixed | |

**Sexual Orientation**

| | |
|---|---|
| Heterosexual | |
| Gay or Lesbian | |
| Bisexual | |
| Not known | |
| Not stated | |
| Person asked and does not know or is not unsure | |

**Alcohol Use**

**How often do you drink alcohol?**

| | |
|---|---|
| Daily | |
| 4 or more times per week | |
| 2-3 times per week | |
| 2-4 times per month | |
| Monthly or less | |
| Never | |

**Daily consumption of alcohol (units)** Click or tap here to enter text.

**Years of alcohol dependence** Click or tap here to enter text.

## Drug Use

### Type

| | |
|---|---|
| Opiates | |
| Cocaine | |
| Crack cocaine | |
| Benzodiazepines | |
| Amphetamines | |
| Cannabis | |

### Frequency

| | |
|---|---|
| Daily | |
| 5-7 days a week | |
| 1-4 days a week | |
| Monthly | |
| Not in the last 6 months | |

**Years of use** Click or tap here to enter text.

### Route of administration

| | |
|---|---|
| Oral | |
| Nasal | |
| Intravenous | |
| Inhaled | |
| Other | |

## Mental Health

**Diagnosis** Click or tap here to enter text.

**Medication** Click or tap here to enter text.

## Current Housing Status

| | |
|---|---|
| Rough sleeping | |
| Sheltered accommodation | |
| Mainstream housing | |
| Accommodation with MH support | |
| Sofa surfing | |

## Prison

| | |
|---|---|
| Has been in prison in the last 12 months | |
| Has been in prison earlier than 12 months ago | |
| Has never been in prison | |

## Disability

| | |
|---|---|
| Behaviour and Emotional | |
| Hearing | |
| Manual Dexterity | |
| Memory or Learning | |
| Mobility and Gross Motor | |
| Self-care | |
| Progressive Conditions and Physical Health | |
| Sight | |
| Speech | |
| Other | |
| Not Stated | |
