# Supplementary material for: Healthcare access and perceived value of liver screening among people experiencing homelessness and substance use disorders: a qualitative study
Source: BMC Health Serv Res. 2025 Dec 6;25:1588. doi: 10.1186/s12913-025-13809-z (PMC12690787; doi:10.1186/s12913-025-13809-z)
Supplement: Supplementary file 2 — Supplementary Material 2: Additional file 2. Interview Topic Guide. The topic guide that was used for the interviews. [file 12913_2025_13809_MOESM2_ESM.pdf]

Additional file 2. Interview Topic Guide

Preamble

Reminder of the purpose of study

Explanation of ethics, consent and confidentiality of interview and analysis

Structure of the interview (may be some overlap in questions and responses)

Introduction

As mentioned, the purpose of this study is to evaluate the implementation of the transient elastography (i.e. Fibroscan^{TM}) pathway for people experiencing homelessness and substance misuse. Transient elastography is an accessible, user-friendly and non-invasive technique for assessing the fibrosis ('scarring') of the liver. Detection and treatment of liver fibrosis are crucial to prevent liver cirrhosis and other serious complications. We want to find out about your experience with this pathway and identify ways in which we can make it better.

TOPIC GUIDE

1. **Referral process to Rough Sleepers Drug and Alcohol Team (RSDA)**

   Prompts:

   - Person/service who referred and other professionals involved in their care
   - Reason for referral to RSDA
   - Reason for referral to transient elastography/barriers to referral
   - Facilitators to referral

2. **Understanding of alcoholic liver disease**

   Prompts:

   - Effects of alcohol on the liver
   - Fatty change/fibrosis/cirrhosis/transplant
   - Signs of alcoholic liver disease e.g. Jaundice, leg swelling
   - Frequent attendances to A+E/Hospital due to alcohol related problems
   - Reversibility and prognosis
   - How important is ALD and its severity to their motivation to drink/not drink?

3. **Previous experience with liver investigations**

   Prompts:

- Blood tests including LFTs, clotting and bleeding risk
- Biopsy/Fibroscan

  – have they ever had a Fibroscan or been offered a scan but not attended
- Reason for referral to these tests
- Location (hospital, GP, specialist service)
- Barriers to testing in the past
- Facilitators to testing in the past

4. **Understanding of these tests**

   Prompts:

- Whether the results of these tests were explained to them
- Who explained these tests (GP, consultant, nurse)
- Methods used to explain the values of these tests
- Whether they were referred to any other service because of these tests

- Whether they were offered any lifestyle advice following these tests

5. **Transient elastography procedure**

   Prompts:

- Person/specialty who performed the examination
- What they liked most about the method (non-invasive, short duration, accessing the location)
- What they liked least about the method
- Person-centred approach in performing the examination
- Person-centred approach in explaining the results (format of results [paper or electronic records] things that would help explain the results more effectively)
- Comparison with other types of liver investigations
- Resources and materials (leaflets, information sheet, useful numbers)
- Do they remember and understand the results of the scan
- Barriers and facilitators to having the scan
- If this intervention would work for everyone

6. **After transient elastography**

   Prompts:

- Whether they were referred to a specialist liver (hepatology) service
- Whether they were offered any lifestyle advice following the results
- What changes have they made since they had the scan
    - E.g. Reduced alcohol intake or not
    - E.g. adopted harm reduction and other strategies to improve the health of the liver
- Have they been offered medically assisted withdrawal e.g. inpatient or community alcohol detoxification
- Whether they have seen changes in their physical health following the examination
- How satisfied are they with the service
- Whether they would recommend this service to their peers

7. **Anything else that the participant would like to add**

-Thank participant and end the interview-
