# Supplementary material for: Healthcare access and perceived value of liver screening among people experiencing homelessness and substance use disorders: a qualitative study
Source: BMC Health Serv Res. 2025 Dec 6;25:1588. doi: 10.1186/s12913-025-13809-z (PMC12690787; doi:10.1186/s12913-025-13809-z)
Supplement: Supplementary file 3 — Supplementary Material 3: Additional file 3. Important People Initial Interview Results. Table detailing the results of the Important People Initial Interview instrument. [file 12913_2025_13809_MOESM3_ESM.docx]

Additional file 3. Important People Initial Interview Results

| n | 11 |
| --- | --- |
| Number of important people listed Mean (SD/range) | 2 (0.6, 1-3) |
| Relationship to participant % |  |
| Parent | 27.3 |
| Spouse | 0 |
| Significant other | 9.1 |
| Child | 36.3 |
| Sibling | 9.1 |
| Other relative | 0 |
| Friend | 9.1 |
| Other | 9.1 |
| Frequency of contact % |  |
| Daily | 18.2 |
| 3-6 times a week | 27.3 |
| Once or twice a week | 27.3 |
| Every other week | 9.1 |
| About once a month | 9.1 |
| Less than monthly | 0 |
| Once in the past four months | 9.1 |
| How important are these people - mean score (SD) | 5 (1.1) “Very important” |
| How supportive they are – mean score (SD) | 4.71 (1.6) “Supportive” |
| Drinking/drug use status % |  |
| Heavy drinker or user | 9.1 |
| Moderate drinker or user | 9.1 |
| Light drinker or user | 9.1 |
| Abstainer | 63.6 |
| In recovery | 0 |
| Don’t know | 9.1 |
| Frequency of important person’s alcohol or drug use % |  |
| Daily | 18.2 |
| 3-6 times a week | 0 |
| 1-2 times a week | 9.1 |
| About every other week | 0 |
| About once a month | 0 |
| Less often than monthly | 0 |
| Once in the past four months | 0 |
| Not in the past four months | 63.7 |
| Don’t know | 9.1 |
| Important person’s reaction to participant drinking/drug use – % |  |
| Encouraged | 0 |
| Accepted | 9.1 |
| Neutral | 36.4 |
| Did not accept | 36.4 |
| Left or made them leave when drinking/using drugs | 18.2 |
| Don’t know | 0 |
| Important person’s feelings about participant coming to treatment % |  |
| Strongly supports it | 81.8 |
| Supports it | 0 |
| Neutral | 0 |
| Mixed | 0 |
| Opposes it | 0 |
| Strongly opposes it | 9.1 |
| Don’t know how they feel about it | 9.1 |
